# SARS-CoV-2 Non-Structural Proteins and Their Roles in Host Immune Evasion

**DOI:** 10.3390/v14091991

**Published:** 2022-09-08

**Authors:** Zheng Yao Low, Nur Zawanah Zabidi, Ashley Jia Wen Yip, Ashwini Puniyamurti, Vincent T. K. Chow, Sunil K. Lal

**Affiliations:** 1 School of Science, Monash University Malaysia, Jalan Lagoon Selatan, Bandar Sunway, Subang Jaya 47500, Malaysia; 2 Infectious Diseases Translational Research Program, Department of Microbiology and Immunology, Yong Loo Lin School of Medicine, National University of Singapore, Kent Ridge, Singapore 117545, Singapore; 3 Tropical Medicine & Biology Platform, Monash University, Subang Jaya 47500, Malaysia

**Keywords:** COVID-19, SARS-CoV-2, immune escape, immune evasion, non-structural proteins, antivirals

## Abstract

Coronavirus disease 2019 (COVID-19) has caused an unprecedented global crisis and continues to threaten public health. The etiological agent of this devastating pandemic outbreak is the severe acute respiratory syndrome-coronavirus-2 (SARS-CoV-2). COVID-19 is characterized by delayed immune responses, followed by exaggerated inflammatory responses. It is well-established that the interferon (IFN) and JAK/STAT signaling pathways constitute the first line of defense against viral and bacterial infections. To achieve viral replication, numerous viruses are able to antagonize or hijack these signaling pathways to attain productive infection, including SARS-CoV-2. Multiple studies document the roles of several non-structural proteins (NSPs) of SARS-CoV-2 that facilitate the establishment of viral replication in host cells via immune escape. In this review, we summarize and highlight the functions and characteristics of SARS-CoV-2 NSPs that confer host immune evasion. The molecular mechanisms mediating immune evasion and the related potential therapeutic strategies for controlling the COVID-19 pandemic are also discussed.

## 1. Introduction

Coronaviruses (CoV) are the primary etiologic agents of the common cold as well as devastating outbreaks and pandemics, including the severe acute respiratory syndrome (SARS), Middle East respiratory syndrome (MERS), and the recent rapidly progressing COVID-19 caused by SARS-CoV-2. As of 31 August 2022, there have been over 600 million cases of COVID-19 associated with about 6.5 million deaths worldwide—the relentless spread of SARS-CoV-2 continues to pose an alarming threat to global public health [1]. Notably, the estimated fatality rate of infection with SARS-CoV-2 (3.4%) is much lower than that of SARS-CoV (9.6%) and MERS-CoV (40%) [2,3]. However, the highly infectious nature and transmission of SARS-CoV-2 contribute significantly to the global and widespread burden of COVID-19. Belonging to the Coronaviridae family, coronaviruses can be generally classified into different genera, namely alpha-, beta-, gamma-, and delta-coronaviruses—of which certain beta-coronaviruses are responsible for severe respiratory illnesses and high fatalities [4].

Apart from the three coronaviruses that caused significant outbreaks or pandemics, there are other human coronaviruses (HCoV) known to cause human diseases, namely HCoV-OC43, HCoV-HKU1, HCoV-NL63, and HCoV-229E [5]. The latter four coronaviruses are far less pathogenic and cause merely mild respiratory infections and the common cold. Although the origin of SARS-CoV-2 is yet to be definitively determined, several pieces of evidence point towards bats as the animal reservoir [6]. Common symptoms of COVID-19 include dry cough, fatigue, headache, fever, dyspnea, and pneumonia—similar to the symptoms of SARS and MERS. Less common symptoms may include dysgeusia, ageusia, diarrhea, and muscle weakness [7].

Being a beta-coronavirus, SARS-CoV-2 shares a similar morphology and structure with its beta-coronavirus counterparts. SARS-CoV-2 shares genetic similarity of 79% and 51.8% compared with SARS-CoV and MERS-CoV, respectively [8]. In general, coronaviruses are a group of enveloped, positive-sense RNA viruses with genome sizes ranging from 26 to 36 kb. Within the envelope, there are four main structural proteins, namely the spike (S), membrane (M), envelope (E), and nucleocapsid (N) proteins—all of which contribute to viral host entry and assembly of virion progeny (Table 1) [9]. The RNA genome possesses a 5′ cap and poly(A) 3′ tail, allowing it to serve as messenger RNA for translation by the host cell machinery. At the 5′ end of the genome resides the open reading frame (ORF) 1a and 1b replicase genes encoding proteins that are subsequently cleaved to yield non-structural proteins (NSPs) 1 to 16. These NSPs encode many crucial enzymes for RNA processing and viral replication, including the RNA-dependent RNA polymerase (RdRp), nidoviral RNA uridylate-specific endoribonuclease (NendoU), and 2′O-methyltransferase (MTase). On the other hand, at the 3′ end of the genome lies the structural proteins (S, E, M, N) with the accessory genes scattered in between [10].

In some patients, COVID-19 can lead to certain severe complications, such as septic shock, multiple organ failure, and the more pronounced acute respiratory distress syndrome [15]. In times of urgent need, drug repositioning represents a feasible approach to combat COVID-19. Also known as drug repurposing, drug reprofiling, or drug recycling, this strategy finds new uses for previously established drugs to treat diseases other than their initially intended indications [16]. The benefits include a shorter drug development time accompanied by lower costs since the repurposed drug has already undergone rigorous safety and pharmacokinetic profiling [17]. Currently, there are several approved antiviral agents against COVID-19 (e.g., remdesivir, molnupiravir), with other drug candidates being reconsidered for drug repositioning [18]. COVID-19 vaccines have also been developed and approved for clinical use, namely ChAdOx1-S (Oxford/AstraZeneca), Ad26.COV2.S (Janssen), mRNA-1273 (Moderna), BNT162b2 (Pfizer BioNtech), BBIBP-CorV (Sinopharm), Coronavac (Sinovac), BBV152 COVAXIN (Bharat Biotech), Ad5-nCoV-S (Cansino), and NVX-CoV2373 (Novavax) [19].

**Figure 1:**
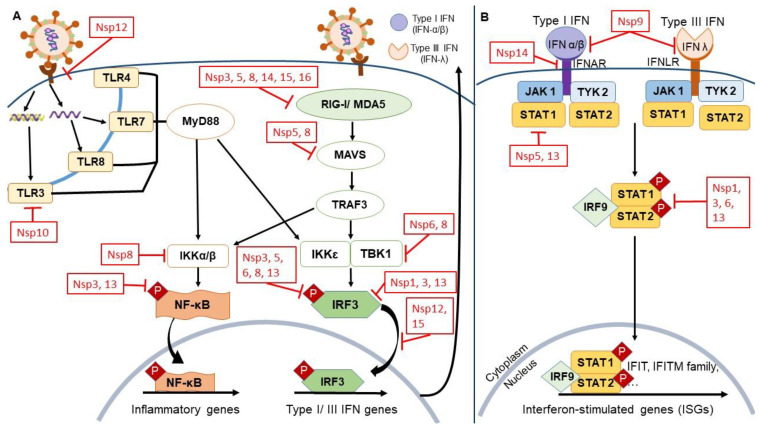
An overview of the SARS-CoV-2 non-structural proteins which contribute to host immune escape (in red boxes). (**A**) Pathogen-associated molecular patterns (PAMPs) of the virus are recognized by various immune cells (macrophages, monocytes, neutrophils, dendritic and epithelial cells). Upon infection, these immune cells recognize foreign viral antigens and molecules (PAMPs) via pattern recognition receptors (PRRs) such as Toll-like receptors (TLRs) and RIG-I-like receptors (RLRs)—thus stimulating cytokines and IFNs, and subsequently inducing host immune responses. (**B**) Type I and type III IFNs are produced and bind to their specific cell surface IFN receptors—thus activating JAK/STAT signaling to promote IFN-stimulated genes (ISGs) to achieve antiviral responses. (Adapted from Park et al. [22]).

However, mass vaccination efforts may easily be thwarted by the emergence of SARS-CoV-2 variants with enhanced transmission and replication which are progressing at accelerated rates [20]. The reduced efficacies of existing vaccines against such new variants therefore render vaccinated individuals at risk of vaccine breakthrough infections [21]. Consequently, the demand for effective antivirals is intensifying due to the increasing daily cases of COVID-19 in many countries. Based on the important virus–host interactions, due consideration and attention should be given to the various structural and non-structural proteins of SARS-CoV-2 that antagonize host interferon (IFN) synthesis and signaling with typically predominant roles in activating the host antiviral responses. These viral proteins allow SARS-CoV-2 to effectively evade the host immune system, leading to increased severity and rapid disease progression. Hence, this highlights the need to better understand the roles of NSPs in viral replication and immune evasion with the aim of discovering feasible targets for drug design and repositioning (Figure 1).

### The Immune Signaling Pathways Associated with Viral Infection

Pathogen-associated molecular patterns (PAMPs) constitute important viral and bacterial molecules that are sensed by specific detection mechanisms of the human immune system. During cellular infection, PAMPs are recognized as foreign viral antigens and molecules via host pattern recognition receptors (PRRs). PRRs include the intracellular nucleic acid sensors associated Toll-like receptors (TLRs) 3, 4, 7, 8; retinoic acid-inducible gene I-like receptors (RLRs such as RIG-I); and melanoma differentiation-associated protein 5 (MDA5) [23]. The activation of these receptors induces downstream signaling molecules such as adaptor proteins MAVS and MyD88 kinases, TANK binding kinase 1 (TBK1), and inhibitor of κ-B kinase ε (IKKε) (Figure 1). These in turn promote the phosphorylation of nuclear factor-κB (NF-κB) transcription factor which produces numerous pro-inflammatory cytokines such as IL-1, IL-6, and TNF-α [24].

Concurrently, adaptor proteins MAVS and MyD88 kinases trigger the phosphorylation of IFN-regulatory factors (IRFs) such as IRF3 and IRF7. This then stimulates the synthesis of IFNs—type I (IFN-α, IFN-β, IFN-ε, IFN-κ, IFN-ω) and type III (IFN-λ) IFNs [23]. Following that, IFN-α/β binds to the respective IFN-α/β receptor (IFNAR1/IFNAR2); the associated JAK1 and TYK2 then phosphorylate cytoplasmic transcription factors STAT1 and STAT2, thereby forming a heterodimer transcription factor complex (STAT1/STAT2/IRF9)—giving rise to IFN-stimulated gene factor 3 (ISGF3) [25]. During viral infection, ISGF3 plays an important role in inducing promoters containing the IFN-stimulated response element (ISRE)—thus upregulating antiviral IFN-stimulated genes (ISGs), including 2′,5′–oligoadenylate synthetase (OAS) and protein kinase R (PKR). Ultimately, this process facilitates host suppression of viral replication [26].

It is noteworthy that SARS-CoV-2 NSPs mainly affect the human innate immune responses, contributing to immune escape. Conversely, SARS-CoV-2 structural proteins, especially spike protein mutations, can acquire different means of adaptive immune escape. For example, one study revealed that K417N and Y155 mutations found in B.1.1.7 and B.1.351 variants significantly diminish the capability of the antigenic peptide to be loaded onto the relevant human leukocyte antigen or HLA-A class I, thus abolishing host CD8+ T cell responses [27]. The objective of this review is to discuss these multiple modes of immune escape exploited by the various NSPs of SARS-CoV-2, and to address potential strategies against specific NSPs in order to counter the severity of COVID-19.

## 2. SARS-CoV-2 NSPs and Their Multiple Mechanisms of Immune Escape

### 2.1. NSP1

NSP1 is the first protein produced by the N-terminal region of SARS-CoV-2. It is characterized by an approximate length of 180 amino acids (aa), a hydrophobic core, and flexibly disordered N- and C-terminal tails [28,29]. Highly conserved in SARS-CoV and SARS-CoV-2, the antiparallel, hydrophobic core domain comprises a seven-stranded β-barrel motif capped by two parallel 3_10_ helices, an additional β-strand, and an α-helix [28]. NSP1 from SARS-CoV-2 shares sequence similarities of 84.4%, 96.7%, and 95.6% with NSP1 of SARS-CoV, RaTG13, and pangolin-CoV, respectively [30]. Despite the high protein sequence similarity of NSP1 of SARS-CoV-2 with SARS-CoV, the latter lacks the 3_10_ helix and additional β-strand in the core domain which are postulated to contribute to protein stability [29,31].

NSP1 plays critical roles in the viral life cycle via immune evasion and translation inhibition. The main underlying mechanism is its interaction with the small ribosomal subunit (40S) and NXF1. The 40S subunit binds to initiation factors that scan mRNAs prior to protein synthesis (shown in Table 2). The C-terminus of SARS-CoV-2 NSP1 directly binds to 40S during the initiation phase of translation [30,32]. The first α-helix of the NSP1 C-terminus interacts with uS3 and uS5 in the head and body of 40S, whereas the second α-helix interacts with the h18 ribosomal RNA (rRNA) of 40S [32]. In addition, the matching surface charge between the α-helix of NSP1, uS3, uS5, and h18, as well as the overlapping shape between the NSP1 C-terminus and mRNA channel allows NSP1 to effectively block the mRNA path [32]. In fact, the NSP1 C-terminus shares a similar structural region and interacting nucleotides with known ribosome inhibitors, SERBP1 and Stm1 [32,33]. Consistent with this, dose-dependent inhibition of translation occurs with increasing concentrations of SARS-CoV-2 NSP1 [30]. Deletion of its C-terminal domain diminishes NSP1 translational inhibitory activity and its ability to bind to 40S [30].

Additionally, SARS-CoV-2 NSP1 interacts with NXF1-NXT1 complex (host mRNA nuclear export factor) which facilitates mRNA translocation to the cytoplasm via the nuclear pore complex (NPC). Such binding inhibits the docking of cargo mRNA at the NPC (Nup153, Nup214, Nup358), resulting in impaired mRNA translocation and translation [34]. Moreover, NSP1 can induce mRNA cleavage via the recruitment of exonucleases (i.e., Xrn1)—particularly mRNAs containing an internal ribosome entry site (IRES) and non-viral capped mRNAs [35]. Interestingly, viral mRNAs can escape mRNA degradation and translation inhibition, although the exact mechanism is unclear. The presence of the stem-loop 1 (SL1) in the 5′-UTR of viral mRNAs may play a role in this evasion by the recruitment of free ribosomes and competitive binding with the ribosome for NSP1 binding [33]. Since a mutation in SL1 abolishes such activity and downregulates viral replication, it is possible that SL1 mainly contributes to the immune escape of SARS-CoV-2 [33,36]. With limited ribosomes, viral mRNAs containing 5′-UTR are favored over less efficient cellular mRNAs, possibly due to the potential “unplugging” of NSP1 from 40S during the initiation of viral translation [37,38]. Highly conserved NSP1 amino acids, 124R/125K, may contribute to the stability of viral mRNAs since mutations on these sites can induce strong antiviral responses and result in decreased viral replication [39,40].

The Immune escape of NSP1 is evident in the downregulation of antiviral IFNs. Various signaling pathways (NF-κB, IRF3/IRF7, JAK/STAT, ATF2/c-Jun) are also affected upon host translation inhibition, leading to exuberant cytokine and chemokine production [41]. SARS-CoV-2 NSP1 acts on IFN-α signaling by attenuating STAT1 and STAT2 phosphorylation and subsequent nuclear translocation—even more efficiently than SARS-CoV [42,43]. The binding of NSP1 to 18S rRNA results in downregulation of IFN-β protein, endogenous IFN-β-responsive mRNAs, IFN-λ1, and IL-8 [32,33]. The expression of luciferase induced by ISRE (found in the promoter regions of most ISGs) is reduced by NSP1 in a dose-dependent manner [32]. An in silico study demonstrated that montelukast sodium hydrate (FDA-approved leukotriene receptor antagonist for asthma) can bind to the C-terminal helices of NSP1 which coincide with the ribosome-binding interface [44]. The six residues (153P, 157F, 160N, 164K, 165H, 171R) within the NSP1 C-terminus involved in drug binding exhibit minimal mutations across SARS-CoV-2 genomes in the databases. Montelukast sodium hydrate can reduce viral spike protein expression and viral copy number, accompanied by diminished virus titer. Moreover, increasing concentrations of montelukast sodium hydrate can rescue NSP1-regulated translational inhibition [44]. Similarly, an anticancer drug, mitoxantrone dihydrochloride (MTX), also interacts with SARS-CoV-2 NSP1 with good binding affinity [45]. MTX has been reported to act as a cell surface heparan sulfate inhibitor that is essential for SARS-CoV and SARS-CoV-2 viral entry, suggesting therapeutic potential [46].

### 2.2. NSP3

In the family of coronaviruses, NSP3 is the largest multi-domain protein in which the domain organization differs between genera. Among the 10–16 domains, eight domains (hypervariable region, macrodomain, ubiquitin-like domain 1 (Ubl1), ubiquitin-like domain 2 (Ubl2), papain-like protease domain (pLpro), ectodomain, Y1, CoV-Y) and two transmembrane regions are highly conserved [47]. NSP3 of SARS-CoV-2 shares 91.8% amino acid sequence similarity with SARS-CoV NSP3 [48]. NSP3 possesses cleaving activities on the NSPs via the pLpro domain, including NSP3 self-cleavage [49]. The pLpro between SARS-CoV and SARS-CoV-2 differ in substrate preference, whereby SARS-CoV pLpro cleaves ubiquitinated substrates, while SARS-CoV-2 pLpro cleaves ISGylated substrates [50,51].

NSP3 exhibits several immune escape mechanisms that facilitate viral replication. For example, Ubl1 located at the NSP3 N-terminus regulates interaction between the viral N protein and single-stranded RNA, whereas Ubl2 is proposed to inhibit IFN production [52,53]. Notably, the primary immune escape mechanism of NSP3 lies in the ability of pLpro to target ISG15 modifications. ISGylation is known to promote JAK/STAT-dependent antiviral responses and virus-dependent IRF-3 degradation, which in turn facilitates type I IFN gene expression [54]. Moreover, ISGylation of viral capsid proteins and cellular factors can disrupt viral particle assembly and budding [55,56]. As such, the removal of ISG15 modification by NSP3 suppresses IFN-I production and impairs host inflammatory responses. SARS-CoV-2 NSP3 possesses de-ubiquitinase activity, thereby removing ISG15 modifications from targeted proteins. Binding of GRL-0617 (a repurposed drug targeting SARS-CoV-2) to NSP3 pLpro domain attenuates the ability of NSP3 to hamper ISG15 modifications [57].

The removal of ISG15 modifications affects multiple signaling pathways, including the MDA5 signaling pathway. MDA5 is essential for viral detection where the binding of viral RNA to the C-terminus and helicase of MDA5 leads to signaling-primed conformation and recruitment of enzymes for MDA5 modification [58]. ISG15 is required for initial MDA5 activation upon detection of viral RNA, in which an immediate innate sensor (i.e., RIG-I) upregulates ISG15 to activate MDA5 [59]. SARS-CoV-2 PLpro can directly inhibit MDA5 ISGylation via dysregulation of MDA5 phosphorylation, culminating in a dampened immune response. Expression of SARS-CoV-2 PLpro decreases ISGylation of cellular proteins, IRF3 and TBK1 phosphorylation, and nuclear translocation of IRF3 [51]. Since IRF3 and TBK1 play crucial roles in type I IFN secretion and the NF-κB pathway, both these immune pathways are inhibited.

The addition of GRL-0617 (known inhibitor of PLpro) to SARS-CoV-2-infected cells results in upregulation of phosphorylated IRF3, TBK1, and p65. Subsequently, IRF3 ISGylation, IFN pathway, and NF-κB pathway are activated. This leads to a dose-dependent inhibition of cytopathic effects, reduction in both active viral replication, and release of viral particles. Moreover, GRL-0617 treatment markedly rescues the expression of IFN-responsive genes such as ISG15, OAS1, PK1, and MX1 [51]. One study revealed that an anti-diabetic drug (sitagliptin) and a hepatitis C virus (HCV) NS5A inhibitor (daclatasvir) can inhibit PLpro activity and decrease SARS-CoV-2 replication [60]. It is noteworthy that diabetic patients on sitagliptin treatment have lower mortality rate (18% versus 37%) and improved clinical outcomes (60% versus 38%) compared to control patients. It is postulated that sitagliptin inhibits viral entry by binding to the catalytic site of PLpro, whereas daclatasvir binds to the allosteric site.

### 2.3. NSP5

NSP5, also known as main protease (M^pro^) or 3C-like protease (3CL^pro^), is a three-domain cysteine protease which is highly conserved amongst coronaviruses [61]. NSP5 possesses two domains (I and II) at the N-terminus which forms a chymotrypsin-like fold consisting of antiparallel β-barrels; whereas the α-helical domain III connects to domain II via a long loop region [62,63]. The interaction between two NSP5 protomers forms the substrate-binding site, Cys-His catalytic dyad, which plays an essential role in the proteolytic processing activity on replicase polyproteins (pp1a and pp1ab) [62,64]. Domain III of two protomers stabilizes the chymotrypsin-like fold and promotes NSP5 dimerization [65,66]. Consistent with the high similarity (96%) between NSP5 of SARS-CoV-2 and SARS-CoV, variations between the two NSP5 structures are minimal by a difference of 12 residues. Notably, Thr283 in between two domain III in SARS-CoV NSP5 is substituted by alanine in SARS-CoV-2 NSP5. Thus, the hydrogen bond between Thr283 residues is abolished, allowing two domain III to have closer contact, leading to slightly increased catalytic efficiency in SARS-CoV-2 compared to SARS-CoV [64]. SARS-CoV-2 NSP5 exhibits a relatively higher inhibitory activity on IFN induction than SARS-CoV NSP5 [67].

Similar to other NSPs, NSP5 possesses several immune escape mechanisms. Studies reveal that NSP5 mainly acts on RIG-I and MAVS to exert such activities. RIG-I activation involves K63-linked ubiquitination mediated by TRIM25 to release CARD domains. NSP5 is able to reduce K63-linked ubiquitination of RIG-I and the association between RIG-I and TRIM25 [67]. Moreover, NSP5 overexpression attenuates the phosphorylation of TBK1 and IRF3, leading to inhibition of IFN-β via the IRF3-mediated and NF-κB-mediated signaling pathways. NSP5 inhibits RIG-I by cleaving a small N-terminal fragment at the Q10 residue, resulting in a smaller RIG-I protein size [68]. Given that the 10 N-terminal amino acids are essential for RIG-I-mediated IRF3 activation, the loss of Q10 residue impairs the ability of RIG-I to interact with free ubiquitin chains and activate IRF3 [69]. In addition, it lowers IFN induction and NF-κB activation in a dose-dependent manner, together with the inhibition of TBK1 and IRF3 phosphorylation [68].

Furthermore, the reduction of MAVS protein by SARS-CoV-2 is documented in COVID-19 patients. Interestingly, MAVS mRNA is elevated upon infection, suggesting that NSP5 reduces MAVS protein independently of its protease activity. Presumably, NSP5 facilitates K48-linked ubiquitination of MAVS and serves as an E3 ligase to degrade MAVS via the ubiquitin-proteasome system. Out of the three MAVS isoforms (MAVS30, MAVS50, MAVS70), NSP5 greatly reduces MAVS70 expression by targeting the K136 residue for ubiquitination and degradation. Both the cleavage in Q10 and K136 residues inhibits RIG-I-MAVS heterodimerization in a dose-dependent manner [68]. The inhibitory effect of NSP5 is also supported by the reduction of MAVS-induced IFN-β production by up to 10-fold [70]. However, it is proposed that NSP5 does not explicitly target IFN-β production since the papain-like protease function of NSP5 is more likely to exhibit biological activities that impair cellular homeostasis. Conversely, another study argued that NSP5 stabilizes MAVS via SUMOylation instead, resulting in NF-κB activation and enhancing the release of cytokines [71]. Stimulated cytokine release subsequently leads to the cytokine storm that occurs in patients with severe COVID-19, contributing to poorer disease prognosis. The knockdown of MAVS and attenuation of SUMOylation can indeed impair NSP5-mediated NF-κB activation and cytokine induction [71,72].

The roles of RIG-I and MAVS in the immune evasion strategies of NSP5 have been further validated by using NSP5 mutants, in which viral proteolytic activity is diminished [73]. Ectopic expression of NSP5-resistant RIG-I-Q10E and MAVS-K136R restores the immune response, and significantly elevates the expression of antiviral genes (IFN-β, ISG56, ISG15, IL-6, IL-8). Upon RIG-I-Q10E and MAVS-K136R expression, viral RNA is reduced up to 90% accompanied by lower virus titers. Similarly, treatment with vinyl sulfone or 2CN115 (a small molecule NSP5 inhibitor that targets the catalytic residue of cysteine proteases) restores the expression of RIG-I and induction of IFN and ISGs. Moreover, 2CN115 reduces NSP5-mediated RIG-I cleavage in a dose-dependent manner. Thus, infectious virions decrease by 100- and 500-fold following treatment with 1 and 4 μM of 2CN115, respectively [68]. Ivermectin, an antiparasitic drug, is reported to interact with the catalytic dyad (Cys145 and His41) of NSP5 via its carbonyl group. Interestingly, the stability and interaction of ivermectin require NSP5 in its homodimeric form. Hence, ivermectin hinders the activity of NSP5 by up to 85%, thereby conferring this drug a new antiviral mechanism [74].

Other immune evasion mechanisms utilized by NSP5 include the inhibition of HDAC2 nuclear transport due to the presence of a cleavage site between nuclear localization sequence and HDAC domain, resulting in impaired HDAC-mediated inflammation and IFN response [73]. The release of type I IFNs can induce transcription of ISGs via JAK/STAT1 signaling. NSP5 promotes the autophagic degradation of STAT1, evident by decreased nuclear translocation and protein levels of STAT1 upon infection. Enzymatically inactivated NSP5 mutant also fails to facilitate the interaction between STAT1 and p62 autophagic receptor [67]. Taken together, the immune evasion of NSP5 serves as an attractive target for antiviral therapy.

### 2.4. NSP6

NSP6 is a 34-kDa protein comprising 290 aa with six transmembrane domains and two small luminal domains. Its overall three-dimensional (3D) structure includes fourteen α-helices, two antiparallel β-strands, sixteen turns, and a highly conserved C-terminus [48,75]. These are located between the third and fourth as well as fifth and sixth transmembrane in the endoplasmic reticulum (ER) lumen [76]. Amino acid sequence alignment of NSP6 from SARS-CoV and SARS-CoV-2 reveals sequence identity and similarity of 88.2% and 98.3%, respectively [48]. Consequently, their functions are perceived to share some similarities. Binding of NSP6 to NSP3 and NSP4 promotes the formation of double-membrane vesicles in infected cells, giving rise to replication-transcription complexes (RTCs) or replication organelles (RO) for viral replication [77,78]. Furthermore, NSP6 confers a protective role over viral production by blocking the development of ER-induced autophagosome/autolysosome vesicles. Specifically, regulation of the ER stress response by SARS-CoV-2 NSP6 is attributed to its interaction with the host sigma receptor [73]. On the other hand, SARS-CoV NSP6 activates autophagy via the omegasome pathway, implying its potential role in facilitating the assembly of replicase proteins or directing immunomodulatory proteins for degradation [79].

For activation of the host innate immune system, one of the ways by which the presence of SARS-CoV-2 double-stranded RNA is detected is via the RLR pathway. Hence, binding of RLRs to viral RNA through a C-terminal helicase domain triggers a conformational change, revealing the N-terminal CARD. Interactions between RLR CARD and CARD on MAVS protein subsequently induces MAVS oligomerization on mitochondrial membranes. Kinase auto-phosphorylation and upregulation of transcription factors IRF3 and NF-κB are initiated via the recruitment of TBK1 or IKKε. The phosphorylation of IRF3 results in homodimerization and nuclear translocation, while IRF3 along with NF-κB promote the expression of type I and III IFN genes and virus-stress inducible genes (VSIGs) [80]. In response to RIG-I CARD in HEK293T human embryonic kidney cells, NSP6 can reduce IRF3 phosphorylation, leading to an inhibitory effect on IFN-β promoter activity. A direct physical interaction also occurs between NSP6 and TBK1, contributing to TBK1 phosphorylation and nuclear translocation of IRF3 [43]. Nonetheless, there is inconsistency in NSP6-TBK1 interaction and NSP6-induced IFN inhibition, suggesting the influence of viral protein expression levels on antiviral immune evasion [42,73].

Typically, in a late host antiviral response, type I and III IFNs bind to cognate cellular receptors to activate the JAK/STAT pathway, leading to phosphorylation of STAT1 and STAT2 transcription factors [81]. Binding of STAT1, STAT2, and IRF9 transcriptional complexes to ISREs then enhances the expression of ISGs with antiviral functions [43]. In particular, the modulation of ISRE promoter activity is augmented under low NSP6 expression levels [42]. Among the highly pathogenic coronaviruses, the inhibition of type I IFN as well as STAT1 and STAT2 phosphorylation by SARS-CoV-2 NSP6 is far more efficient compared to the NSP6 of SARS-CoV and MERS-CoV [43].

Knowledge on immune evasion mechanisms by NSP6 has led to its consideration as an antiviral target. In a transcriptome analysis of SARS-CoV-2-infected lung epithelial cells and COVID-19 patient lung tissues, NSP6 upregulation is correlated to inflammatory cell death via NLRP3/ASC-dependent caspase-1 activation, IL-1β/18 maturation, and lung epithelial cell pyroptosis. The direct interaction of NSP6 with ATP6AP1 (a vacuolar ATPase proton pump component) prevents its cleavage-mediated activation—hence causing suppression of lysosome acidification. Furthermore, treatment with 1α,25-dihydroxyvitamin D3 (active form of vitamin D3), metformin (anti-diabetic), or polydatin (phytochemical) can attenuate NSP6-induced autophagic flux impairment, inflammasome activation, pyroptosis, and reactivate oxygen species (ROS) production [82]. An in silico analysis further suggests that dextromethorphan (sigma receptor agonist) leads to NSP6 destabilization and an increase in conformational dynamics, whereas haloperidol (anti-psychotic) confers stronger binding affinity and favorable molecular interactions [75].

### 2.5. NSP7

Monomeric NSP7 is a 9-kDa protein comprising approximately 80 aa [48,83]. The 3D structure of NSP7 is available as a complex with NSP8 in the dimeric, linear heterotetrameric or cubic heterotetrameric conformation. One of the seven structures of the complex has been published [83]. High-resolution crystal structures of the heterodimeric form of NSP7-NSP8 indicate that NSP7 has a three-helical coiled-coil bundle comprising three consecutive α-helices, followed by a C-terminus appearing as a short, not well-defined helix [84]. Comparative structure-based sequence analyses of NSP7 from SARS-CoV-2 with SARS-CoV and feline coronavirus (FCoV) show sequence identity of 99% and 42%, respectively [48,85]. Highly conserved interface residues in NSP7–NSP8 suggest that the interaction mechanism for the complex formation is similar across coronaviruses [85]. Along with NSP8, NSP7 functions an essential cofactor that binds to NSP12, forming a complex which stabilizes the polymerase domain—this facilitates template recognition and nucleotide polymerization [86].

Despite NSP7 being a cofactor of NSP8, both have distinct mechanisms of immune evasion. Research on NSP7 has focused more on its contribution to disease pathogenesis rather than immune evasion. Significant suppression of IFN-α signaling is observed in SARS-CoV-2 NSP7-transfected HEK293T cells [42]—this is consistent with the antagonism of IFN signaling by SARS-CoV NSP7 [52].

Instead of NSP7 alone, its formation as a complex with NSP8 and NSP12 is proposed to serve as a superior antiviral target. A virtual screening of drug candidates against this complex shortlisted eight compounds, namely cepharanthine, nilotinib, filibuvir, lonafarnib, olysio, saquinavir, tegobuvir, and tipranavir [87]. Isolated from *Stephania tetrandra*, cepharanthine is an anti-cancer, anti-inflammatory, anti-parasitic, and antioxidant alkaloid tetrandrine that targets both the NSP7–NSP12 and NSP8–NSP12 interface of SARS-CoV-2, in addition to the NSP8–NSP12 interface of SARS-CoV [87,88]. Lonafarnib (a non-peptidomimetic inhibitor of farnesyltransferase) and nilotinib (a tyrosine kinase inhibitor) exhibit interaction with the NSP7–NSP12 interface of SARS-CoV-2 [87,89,90]. Moreover, filibuvir (an oral non-nucleoside HCV NS5b RNA-dependent RNA polymerase or RdRp inhibitor), and olysio (a HCV NS3/4a protease inhibitor) can block the NSP8-NSP12 interface of SARS-CoV and SARS-CoV-2 [87,91,92]. Although saquinavir (a human immunodeficiency virus or HIV protease inhibitor) and tipranavir (a non-peptide protease inhibitor) can bind to the NSP7–NSP12 interface of SARS-CoV, their antiviral potential against SARS-CoV-2 remains to be elucidated [87,93,94].

### 2.6. NSP8

In its monomeric form, NSP8 is a 24-kDa protein comprising 198 aa [48,83]. In the heterodimeric NSP7–NSP8 complex, NSP8 has an N-terminus with a highly positively charged α-helix for RNA-binding, followed by another α-helix connected via a long loop to a half β-barrel-like domain consisting of five antiparallel β-strands [84]. A small α-helix is further inserted between the first two strands, in addition to a long loop containing two half-turn helices. Altogether, the conformation of NSP8 resembles a golf club. Comparative structure-based sequence analyses of NSP8 from SARS-CoV-2 with SARS-CoV and FCoV indicate sequence identity of 98% and 41%, respectively [48,85]. Apart from the role of NSP8 as a cofactor with NSP7 to bind to NSP12 as a viral polymerase complex, NSP8 is critically important in extending the template RNA-binding surface. The disordered N-terminal regions are hypothesized to act as molecular handles for recruitment of additional viral factors and organization of the viral replication complex [86].

Compared to NSP7, the mechanisms by which NSP8 evades the host immune response are better understood. In HEK293T cells, NSP8 can suppress MAVS-dependent antiviral responses [95]. In contrast to NSP6, NSP8 preferentially regulates MDA5-mediated responses as opposed to the RLR pathway [42,95]. Binding of NSP8 to MDA5 via its CARD domains causes disruption of MDA5–MAVS signalosome formation by impairing its K63-linked polyubiquitination—this subsequently inhibits any MDA5-mediated immune responses. These include the downregulated expression of ISGs, as well as reduced secretion of type I IFNs (IFN-β), pro-inflammatory cytokines (IL-6, CCL-20), and other immune factors (TNF-α, IFIT1, IFIT2). Similar to NSP6, the overexpression of NSP8 induces a reduction in TBK1 phosphorylated levels, followed by inhibition of phosphorylated IRF3 and IKKα/β. This results in the stabilization of IκBα, leading to inhibition of NF-κB and p65 phosphorylation [95]. However, it is notable that the suppression of IFN-α signaling by NSP8 is not as significant as other SARS-CoV-2 NSPs such as NSP1, NSP6, NSP7, and NSP14 [43]. Altogether, NSP8 acts as an innate immune suppressor which favors viral replication and transcription.

Similar to NSP7, NSP8 may serve as a potential target for COVID-19 treatment in view of its complex formation with NSP7 and NSP12. Essentially, targeting this complex will be able to block viral polymerase activity.

### 2.7. NSP9

NSP9 is one of the proteins generated by the SARS-CoV-2 ORF1a gene and is thought to be involved in viral RNA synthesis. Structural analyses of SARS-CoV NSP9 show that this protein is dimeric, and forms a single, nucleic acid-binding site for efficient viral replication [96,97]. SARS-CoV NSP9 structure consists of a central core of a six-stranded barrel surrounded by a C-terminal helix and N-terminal extension. RNA binding function is achieved through the β-barrel loop structure, while dimerization and interaction with other proteins are likely conserved through the C-terminal β-hairpin and helix across different coronaviruses [97]. NSP9 of SARS-CoV-2 shares about 97% similarity with SARS-CoV, suggesting functional conservation [98]. One motif that is primarily conserved is the antiparallel, α-helical GXXXG motif at the protein–protein interface, implying that disruption of key residues within this motif may result in diminished RNA binding [99,100].

NSP9 can interact with NSP12 to form part of the RTC which is important for viral replication. This occurs via the insertion of the NSP9 amino terminus to the NSP12 NiRAN domain [98]. NSP10 and NSP14 may also be recruited [99]. NSP9–NSP12 interaction is suggested to act either as a nucleotidylation inhibitor during mRNA-cap formation, or with NSP9 acting specifically as the major substrate for that reaction [101,102]. NSP9 binds to specific sites in the S domain of the 7SL RNA scaffold of signal recognition particle (SRP) and suppresses membrane protein trafficking in cells infected with SARS-CoV-2. Since IFN response is also dependent on SRP, its suppression via NSP9 subsequently decreases IFN response. In addition, protein interaction between NSP9 and the start codon of mRNA encoding COPS5 may play a role in impeding protein translation [33]. NSP9 of SARS-CoV-2 can interact with and impair a component of NPC, nucleoporin 62 (NUP62), thus hindering the translocation of p65 upon TNF-α stimulation [103].

NSP9 is essential to the viral replication process since its deletion may impair RNA synthesis and viral infectivity [104,105]. A mutation of G104E within SARS-CoV NSP9 can alter its protein structure, and arrest viral replication [106]. Furthermore, the dimeric form of NSP9 that is crucial for its functionality is maintained by the conserved GXXXG motif [107]. All these criteria render NSP9 a suitable target to design drug candidates. A structure-based drug repurposing study against SARS-CoV NSP9 replicase revealed certain molecules as NSP9 inhibitors, such as conivaptan, telmisartan, and phaitanthrin D [108]. In addition, other potential NSP9 regions for antiviral targets include the side chains N33, M101, and S105, since they closely resemble protein-binding motif and RNA-binding, glycine-rich regions such as β2-3 and β3-4 [109]. An in silico screening of FDA-approved drugs against NSP9 highlighted five drugs with high affinity to NSP9 replicase—fluspirilene, troglitazone, alvesco, dihydroergotoxinevodart [110]. Further in vivo studies are warranted on such potential drug candidates as inhibitors against SARS-CoV-2 NSP9.

### 2.8. NSP10

NSP10 is one of the proteins which is also implicated in the viral replication process [111]. The structure of NSP10 of SARS-CoV consists of a single domain protein of a coil-rich C-terminus, N-terminal helices stacked against β-sheets, and two zinc fingers [112]. The zinc finger motifs on NSP10 are conserved within the coronavirus family, therefore suggesting the importance of their biological function [113]. SARS-CoV-2 and SARS-CoV NSP10 structures are very similar with only two amino acid changes—P23A at the end of connecting helix loops H1 and H2; and R113K at the apex of α-helix H6 [114]. The structure of NSP10 is unique to the family of coronaviruses as there are no known similar structures in prokaryotes and eukaryotes [114]. An in vitro study revealed that NSP10 localizes in vesicular structures in the perinuclear region of infected cells, further validating its role in viral replication [115].

NSP10 interacts with NSP14 and NSP16, indicating its involvement in the regulation of MTase activity [116,117]. NSP10 acts as a costimulatory protein as it enhances the function of NSP14 exoribonuclease (ExoN) activity by more than 35-fold and does not interfere with N7-MTase activity [111,118]. Indeed, a mutant of NSP10 with loss of its function to bind to NSP14 is associated with inactivation of ExoN activity [111]. Interestingly, NSP16 requires NSP10 binding for it to be active for the formation of RTCs [119]. In vitro studies show that the NSP10–NSP16 complex provides the 2′-O-MTase activity required for NSP14-mediated N7-guanine methylation [120]. The formation of a 2′-O-methylated cap is also critical for immune evasion via the bypassing of IFN-mediated antiviral responses [121]. Furthermore, the interacting sites of NSP10 with both NSP14 and NSP16 overlap [122,123]. However, NSP10 is produced at 3- to 6-fold excess compared to NSP14 and NSP16, allowing both complexes to exist simultaneously [124]. There are a few reports on the interaction of NSP10 with other proteins, and its influence on the host immune response. An in silico study revealed that NSP10 mRNA can bind to TLR3, forming a very stable mRNA complex [125]. This implicates NSP10 mRNA as a PAMP, thus alluding to its role in downstream pro-inflammatory cytokine responses. Overall, NSP10 is deemed important in the replication and transcription process, with the interaction and regulation of these specific proteins [111].

Since these crucial interactions are highly conserved amongst the coronavirus family, NSP10 binding sites could be targeted for antiviral drug design [126]. A virtual screening of FDA-approved drugs identified a number of drugs that may potentially occupy the interacting interface of NSP10–NSP16 or impede the formation of the complex [127]. The list of these drugs and their known functions are summarized in Table 3. Another study identified NSP10–NSP14 inhibitors in synergy with remdesivir, and three drugs were proposed as potential pan-coronavirus therapeutic options [128]. These compounds are isobavachalcone, 2-hydroxyisoquinoline-1,3(2H,4H)-dione (HID), and sofalcone [128,129]. Future investigations and relevant trials could be conducted to further evaluate such potential drug candidates for treating SARS-CoV-2 infection.

### 2.9. NSP12

NSP12, which is also known as the viral RdRp, contains the catalytic activity essential for viral replication [130,131]. NSP12 consists of 932 amino acids, and its structure comprises an N-terminal nidovirus RdRp-associated nucleotidyltransferase (NiRAN) domain which is conserved across the coronavirus family, and a C-terminal right-hand RdRp domain [132]. Other highly conserved surfaces include the template entry, template-primer exit, NTP tunnels, and polymerase-active site as revealed by sequence analyses across the coronavirus family [86]. NSP12 possesses little activity on its own and requires cofactors NSP7 and NSP8 for RNA synthesis activity [133]. NSP7 and NSP8 act as primases and stabilize the RNA-binding region of NSP12 for SARS-CoV-2 genome replication [131,134]. NSP12 of SARS-CoV and SARS-CoV-2 is 96–98% similar—therefore, their structure and function are likely to be identical [135]. However, RNA synthesis of SARS-CoV-2 by the NSP12–NSP7–NSP8 complex has lower efficiency compared to the SARS-CoV equivalent [136]. Furthermore, thermostability properties of SARS-CoV-2 NSP8 and NSP12 subunits are lower than the SARS-CoV counterparts, suggesting evolution of the virus that favors the human host. In addition to NSP7 and NSP8, NSP12 also interacts with NSP5, NSP9, and NSP13 [86].

Some reports support the role of NSP12 in attenuating type I IFN production by inhibiting IRF3 nuclear translocation [43,137,138,139]. However, there are also contradictory studies which show that NSP12 does not suppress IFN-β activation [140]. Therefore, the role of NSP12 with respect to type I IFN is debatable, and more detailed studies are warranted. One study revealed the interaction of NSP12 with receptor-interacting serine/threonine-protein kinase 1 (RIPK1), a protein that mediates innate immune responses such as inflammation and cell death [73]. This was further validated by another study showing that NSP12 promotes RIPK1 activation which stimulates ACE2 and EGFR receptors for viral replication [141]. Amongst all SARS-CoV-2 proteins, NSP12 has the largest number of IgM epitopes, and strongest IgM antibody peptide-based immunogenicity (PBI) [142]. Interestingly, these IgG and IgM epitopes residing on NSP12 are associated with non-survival of COVID-19 patients. Other proteins such as ribonucleoprotein 4B, CREB-regulated transcription coactivator 3, E3 ubiquitin protein ligase, ubiquitin-associated protein 2, PLAKHA5, LARP4B, and CRTC3 also interact with NSP12 [131,143].

Since NSP12 is essential for viral replication, drug candidates that target this region are a focus of attention [144]. Thus, FDA has approved remdesivir for emergency use to treat COVID-19. This broad-spectrum antiviral agent is classified under nucleotide and nucleoside analogs that inhibit polymerases such as RdRp [145]. While this class of drugs may be effective in RdRp inhibition, mutations arising in the NSP12 gene region can lead to loss of drug efficacy and increased resistance. One study into the NSP12 gene found that a common P214L mutation as well as other RdRp mutations can lead to higher rates of mutations of other genes [146]. A virtual drug screening was performed to identify drug candidates with antiviral activity against interfacial pockets of the NSP12–NSP7–NSP8 complex [87]. This screen revealed eight compounds that may be effective for SARS-CoV-2 treatment and requires further evaluation for their antiviral effects. An in silico screening identified the methycobalamin form of vitamin B12 which may inhibit NSP12 [147]. Further research into designing inhibitors against NSP12 would be beneficial as it is a highly conserved protein which may lead to the development of broad-spectrum inhibitors against the coronavirus family [148].

### 2.10. NSP13

NSP13 is a 67-kDa highly conserved RNA triphosphatase (RTPase) enzyme or helicase, a vital coronavirus enzyme that mediates hydrolysis of the 5′ γ-phosphate of the nascent mRNA transcript [149]. This process is crucial for production of the 5′-ppN end for the transfer of guanidine monophosphate by RNA guanylyltransferase (GTase) which is critical for formation of the primary cap structure (GpppN) [126]. NSP13 acts in tandem with NSP10, NSP14, and NSP16 to ensure efficient mRNA capping of SARS-CoV-2. Although its atomic structure is still not fully understood; SARS-CoV-2 NSP13 possesses extremely high sequence identity of 99.8% to SARS-CoV NSP13 helicase. Notably, NSP13 in SARS-CoV-2 differs from SARS-CoV by only one amino acid, I570V. On the other hand, SARS-CoV-2 NSP13 exhibits lower sequence identity of 72% compared to its MERS-CoV counterpart [150]. Akin to SARS-CoV, NSP13 in SARS-CoV-2 is reported to confer immune escape in COVID-19.

Upon viral infection, viral RNA activates the host innate immune response via PAMP receptors such as RLRs and TLRs (Figure 1). The binding of viral RNA to RIG-I/MDA5 then initiates MAVS adaptor protein, phosphorylating TBK1 and IKKε kinases that induce phosphorylation of IRFs which promote production of type I and III IFNs. Next, IFNs bind to their receptive receptors, initiating the JAK/STAT signaling pathway that facilitates downstream antiviral ISGs, culminating in the suppression of viral replication. Being a precursor to SARS-CoV-2 mRNA capping, NSP13 contributes to evasion of RLR recognition, leading to inhibition of NF-κB and IRF3 activation to downregulate the antiviral response. Interestingly, NSP12 is known to play a role in enhancing NSP13 [151]. Studies point towards the NTP-dependent 5′–3′ direction unwinding reaction by NSP13 helicase to convert duplex oligonucleotides into single strands. Thus, there is interest in repositioning of drugs that target ATP binding or direct NTPase activity, inhibition of helicase translocation, e.g., benzotriazole, imidazole, quinoline [152].

Studies reveal a distinct correlation between NSP13 and recognition receptor-associated immune escape. For instance, NSP13 significantly inhibits IFN response in HeLa cells mediated by Sendai virus (SeV), with 30% reduction of luciferase activity in an ISRE-driven luciferase reporter assay. In addition, a two-fold reduction in luciferase activity is observed in the SeV-mediated NF-κB promoter setting. Furthermore, in TBK1-transfected cells, phosphorylated NF-κB expression levels are greatly reduced in the presence of SARS-CoV-2 NSP13. NSP13 can also inhibit NF-κB nuclear translocation. Notably, protein expression of ISGs (e.g., IFIT1) decreases in a dose-dependent manner with respect to increasing amounts of NSP13 [153]. These studies reaffirm that SARS-CoV-2 NSP13 contributes to RLR immune sensor escape, via its critical role in inhibiting immune sensors to attenuate JAK/STAT-mediated antiviral responses, leading to disease establishment in COVID-19 [153,154]. In view of the high homology of NSP13 between SARS-CoV and SARS-CoV-2, drugs targeting NSP13 could be investigated to counter host immune escape in COVID-19. For instance, cepharanthine is an anti-inflammatory drug for treating certain diseases (including leukopenia, alopecia), and displays inhibitory activity against SARS-CoV-2 NSP13 ATPase, implying its potential as an NSP13 inhibitor against COVID-19 [150,155,156]. Several RNA helicase inhibitors against positive single-strand RNA viruses (such as flaviviruses, SARS-CoV) may also have potential inhibitory activity against SARS-CoV-2 NSP13. Examples include benzotriazole, imidazole, imidazodiazepine, phenothiazine, quinoline, anthracycline, triphenylmethane, tropolone, pyrrole, acridone, and bananin derivatives [152,157].

### 2.11. NSP14

NSP14 is a highly conserved NSP known for its 3′ to 5′ ExoN activity and guanine–N7–MTase activity, with the latter mediating RNA capping in tandem with NSP12, NSP13, and NSP16 [139,158]. Akin to NSP16, NSP14 is a S-adenosylmethionine (SAM)-dependent MTase that utilizes SAM as a methyl donor which is essential for the viral life cycle [118,126]. ExoN is an enzyme responsible for RNA degradation via the removal of nucleotides from the 5′ end or the 3′ end of an RNA structure—a critical feature for the synthesis of multiple RNAs from the RNA template in RNA viruses [159]. In particular, the N-terminal ExoN domain in SARS-CoV-2 is predicted to possess proofreading activity that removes mismatched nucleotides introduced by the RdRp [160]. In the presence of NSP10 as a cofactor, it forms an NSP10-NSP14 complex with enhanced ExoN activity [124]. SARS-CoV mutations that affect the NSP10-NSP14 interaction have proven to be lethal to the virus—further highlighting the importance of NSP10 as a cofactor for NSP14 ExoN activity [111]. SARS-CoV-2 NSP14 possesses 99% and 77% amino acid sequence identity with SARS-CoV and MERS-CoV, respectively [158]. Studies suggest that NSP14 is capable of interfering with the host innate immune response to facilitate immune escape in COVID-19.

NSP14 N7–MTase functions in tandem with NSP12, NSP13, and NSP16 by methylating guanine at the N7 position, forming a cap-0 structure (m7GpppA) at the initially generated RNA strand. The initial cap-0 structure (but not the cap-1 structure) can be recognized by the MDA5 RLR sensors that trigger antiviral response genes (such as ISGs and IFITs). At the final capping process, cap-1 production is dictated by NSP16 via methylation of ribose in the 2′-O position, thus, contributing to the overall evasion of viral RNA detection [161]. Furthermore, NSP14 is postulated to inhibit IFNAR1 expression by targeting this receptor for lysosomal degradation—since IFNAR1 is a crucial receptor for the activation of JAK/STAT signaling, this hinders downstream antiviral responses [162]. NSP14 ExoN and N7-Mtase activities are also capable of inhibiting host mRNA translational activity via transcription inhibition. Similar to NSP1, type I IFN-dependent ISG induction is abrogated, compromising the production of antiviral proteins [158]. SARS-CoV-2 NSP14 is a potent translation inhibitor in HEK293T cells (as revealed by O-propargyl-puromycin (OP-Puro) labeling assay), with a significant 75% reduction compared to the empty vector control. The OP-Puro assay is a sensitive and rapid technique to detect cellular protein synthesis via fluorescence microscopy or flow cytometry. The translation inhibition is also shown by the puromycin incorporation assay via immunoblotting using anti-puromycin antibody against OP-Puro-labeled HEK293T cells. Interestingly, NSP14 mutants of both ExoN activity and N7–Mtase do not exhibit any significant translation inhibition, suggesting the requirement for both catalytic activities for successful translation inhibition. NSP10 co-expression significantly increases NSP14 levels, accompanied by augmented translation inhibition activity by wild-type NSP14 (about 2-fold by OP-Puro assay), further emphasizing that NSP10–NSP14 interaction is vital for translation inhibition activity [158]. In the presence of NSP14, the stimulation of IFN-α and IFN-β is strongly repressed, accompanied by significantly diminished endogenous levels of IFNAR1 [162]. Notably, this process can be reversed by addition of bafilomycin A1 (an inhibitor of vacuolar H^+^-ATPase that blocks late-phase autophagy), further proving that NSP14 mediates IFNAR1 degradation via autophagosomal lysosomal activity. Given the high homology of NSP14 between SARS-CoV and SARS-CoV-2, it is useful to target the host immune escape mechanism of SARS-CoV-2 NSP14 by means of drug repurposing. For example, ritonavir (an approved protease inhibitor used to treat HIV/AIDS) can inhibit formation of the complex of NSP14 with RNA –hence preventing excision of nucleotides from the 3′ end of the growing RNA strand, effectively terminating genomic replication [163,164]. In addition, SAM competitive inhibitors or SAM analogs (e.g., DS0464 and SS148) for SARS-CoV demonstrate bisubstrate competitive inhibition of NSP14 activity in SARS-CoV-2 [165,166].

### 2.12. NSP15

NSP15 is conserved amongst members of the coronavirus family [167]. It is also known as a NendoU enzyme under the EndoU family vital for RNA processing during viral replication [168]. In the presence of manganese as cofactor, NSP15 preferentially cleaves RNA nucleotide at the 3′ end of uridylates, producing a 2′-3′ phosphodiester and 5′-hydroxyl termini [169,170]. NSP15 of SARS-CoV and MERS-CoV form hexamers composed of three dimers, similar to SARS-CoV-2 [171]. SARS-CoV-2 possesses sequence identity of 88% and 50% with SARS-CoV and MERS-CoV, respectively [171,172]. Given the high homology of NSP15 between SARS-CoV and SARS-CoV-2, NSP15 is thought to interfere with host innate immune responses, contributing to immune escape in COVID-19.

The RNA virus replicates via a double-stranded RNA (dsRNA) intermediate. These dsRNA intermediates are flagged as “non-self” by several human dsRNA sensors on cells (e.g., macrophages, dendritic cells, monocytes, neutrophils, epithelial cells). Known as PAMPs, these foreign viral molecules trigger the host innate immune system [58,173]. PAMPs are recognized by PRRs which include TLRs and RLRs [15,174]. These interactions activate NF-κB to produce pro-inflammatory cytokines and IRFs that generate type I and III IFNs [175]. Subsequently, antiviral ISGs are expressed to mediate antiviral action via the JAK/STAT signaling pathway [176,177]. Studies demonstrate a strong correlation between NSP15 and downregulation of the downstream antiviral immune response pathway. A model of mutated NSP15 (unstable or catalysis-deficient) coronavirus replicates significantly slower, accompanied by more rapid cell death, in comparison to the wild-type virus [178]. Interestingly, studies also show enhanced phosphorylation of eIF2α (PKR marker), followed by intense type I IFN activation, and elevated caspase-3/7 production (cell apoptosis marker) during infection with NSP15-mutated or NSP15-deficient virus compared to wild-type virus—thus implicating NSP15 in abrogating ISG activation [178,179]. Furthermore, SARS-CoV-2 NSP15 can also prevent IRF3 nuclear localization, which hampers the production of type I and III IFNs [131,139].

SARS-CoV NSP15 can evade MDA5 immune recognition by cleaving its highly conserved RNA structure at the unpaired uridylate bases within the 3′ untranslated region, thereby implying NSP15 as a suitable drug target in the context of SARS-CoV-2 immune escape [180]. NSP15 acts by hydrolyzing the phosphodiester bonds at the uridine sites of the RNA strand, and limits the cellular accumulation of polyU-containing sequences which would normally be detected by MDA5 [181]. Given the high homology of NSP15 between SARS-CoV and SARS-CoV-2, it is warranted to investigate the potential inhibitors of SARS-CoV NSP15 in the quest to develop novel antiviral drugs against COVID-19. Studies propose the potential of certain phytochemicals as possible alternative NSP15 inhibitors. For instance, sarsasapogenin, ursonic acid, curcumin, ajmalicine, novobiocin, silymarin, and aranotin exhibit inhibitory activities against NSP15, thus implying potential inhibition of SARS-CoV-2 replication [182]. Natural compounds such as glycyrrhizic acid (liquorice) have been reported for their activity against influenza, HIV, hepatitis C virus, coxsackievirus, and SARS-CoV [183,184]. Other natural compounds such as epigallocatechin gallate (green tea extract) have also been reported to restrict the proteolytic activity of SARS-CoV 3CLPro/Mpro, effectively reducing the viral load. Notably, epigallocatechin gallate and glycyrrhizic acid can exert NSP15 inhibitory activity via competitive binding with the NSP15 active site [181,185].

### 2.13. NSP16

NSP16 contains a highly conserved enzymatic groove—the KDKE catalytic pocket corresponding to the K6839, N6928, K6968, and Q7001 amino acid residues [186,187]. NSP16 is also known as the SAM-dependent 2′O-MTase that mediates mRNA capping in coronaviruses [188]. In the presence of NSP10 as a stimulatory cofactor, NSP16 preferentially methylates mRNA (m7GpppNm) at the 5′-end, producing capped mRNA [189]. SARS-CoV-2 NSP16 displays high homology to its beta-coronavirus counterparts, SARS-CoV and bat-related SARS-CoV (bat-rSARS-CoV). SARS-CoV-2 NSP16 possesses 93% and 99% amino acid identity to SARS-CoV and bat-CoV-RaTG13, respectively. Notably, the amino acid sequence of the NSP10 cofactor also exhibits a close identity of 100% and 99% to bat-CoVRaTG13 and SARS-CoV, respectively [126].

In mammalian cells, to prevent self-recognition by cytoplasmic RIG-I-like immune receptors (e.g., RIG-I, MDA5), eukaryotic mRNA typically undergoes a series of capping modifications. This entails a series of enzymatic reactions that lead to a 5′ cap-defining structure by RNA polymerase II. Typically, eukaryotic mRNA has a methylated 5′ cap at the N7-guanine position (cap-0), at the ribose-2′-O position of the 5′-penultimate residue (cap-1), and occasionally at adjoining residues (cap-2) [190,191]. NSP16 is responsible for mRNA capping, contributing to immune escape primarily by preventing immune sensor detection during SARS-CoV-2 infection. The entire mRNA capping process by SARS-CoV-2 not only requires the NSP10-activated SAM-dependent NSP16, but also a sequential enzymatic process involving NSP13 and NSP14. This process begins with RNA triphosphatase (RTPase encoded by NSP13) for the hydrolysis of the γ-phosphate of nascent mRNA transcripts, followed by RNA guanylyltransferase (GTase) for the formation of the primary cap structure (GpppN) [186]. Subsequently, N7-guanine-MTase (encoded by NSP14) methylates guanine at the N7 position, forming a cap-0 structure (m7GpppA). The final capping mechanism is then initiated by NSP16 which further methylates at the ribose 2′-OH, giving rise to a eukaryotic mRNA-like cap-1-structure (m7GpppAm). This results in the absence of MDA5 and RIG-I receptor sensor detection, culminating in the downregulation of IFIT to diminish host antiviral responses [192].

Studies illustrate a strong correlation between NSP16 and recognition receptor-associated immune escape. Thus, coronavirus-bearing NSP16 mutants are non-pathogenic in infected wild-type mice. Conversely, viral replication is restored in infected mice lacking MDA5 immune sensors [193]. Furthermore, one study revealed strong repression of IFN-responsive genes upon addition of spliceosome inhibitor molecules and co-expression of NSP16, suggesting that NSP16 binds to the spliceosome component. Hence, mRNA splicing is inhibited, leading to the arrest of immune cell development that further suppresses host innate immune responses [33]. Given the high homology of NSP16 between SARS-CoV and SARS-CoV-2, it is worthwhile to further explore this aspect of immune escape. Sinefungin (a pan-methyltransferase inhibitor) can bind to the active SAM-binding pocket of the NSP10-NSP16 complex to decrease SARS-CoV and SARS-CoV-2 infection levels—thus reiterating the significance of NSP16 in host immune escape [117,193]. In silico analyses suggest that maraviroc and raltegravir (anti-HIV medications) may also have considerable ability to block the entry of the NSP16 active site at the SAM-binding groove, thus alluding to a potential antiviral strategy against COVID-19 [194,195].

**Table 2:** Summary of functions of SARS-CoV-2 non-structural proteins (NSPs) and their modes of immune escape.

NSP	Mode of Immune Escape	References
NSP1	Interacts with 40S subunit to inhibit translation Interacts with the NXF1-NXT1 complex to hinder mRNA translocation to the cytoplasm Recruits exonuclease to induce mRNA cleavage Possible evasion by stem-loop 1 (SL1) via recruitment of free ribosomes; SL1 competitively binds with ribosomes for NSP1 binding Favors translation of viral mRNAs containing 5′-UTR Possibly unplugs from 40S during viral translation R124/K125 stabilizes viral mRNAs	[30,32,33,34,35,37,38,39,40]
NSP3	Removes ISG15 modifications on targeted proteins via PLpro domain Inhibits MDA5, IRF3, and TBK1 phosphorylation Impairs JAK/STAT, MDA5, NF-κB, and IFN signaling pathways	[51,54]
NSP5	Inhibits K63-linked ubiquitination of RIG-I Inhibits interaction between RIG-I and TRIM25 Induces cleavage of Q10 residues to impair RIG-I-mediated IRF3 mediation Attenuates phosphorylation of TBK1 and IRF3 Promotes K48-linked ubiquitination of MAVS, and acts as E3 ligase to degrade MAVS Inhibits HDAC2 nuclear transport Autophagic degradation of STAT1	[67,68,69,73]
NSP6	Interacts with TBK1 for TBK1 phosphorylation and nuclear translocation of IRF3 Reduces IRF3 phosphorylation to inhibit IFN-β promoter activity Low expression levels enhance promoter activity of ISREs Inhibits IFN-1 production as well as STAT1 and STAT2 phosphorylation	[42,43,73]
NSP7	Suppresses IFN-α signaling	[43]
NSP8	Suppresses MAVS-dependent antiviral responses Binds to MDA5 via the caspase activation and recruitment domains (CARD) to interfere with its K63-linked polyubiquitination Disrupts MDA5-MAVS signalosome formation Inhibits MDA5-mediated immune responses Downregulates expression of ISGs and secretion of type I IFNs (IFN-β), pro-inflammatory cytokines (IL-6, CCL-20), and other immune factors (TNF-α, IFIT1, IFIT2) Reduces phosphorylation of TBK1, IRF3, IKKα/β Stabilizes IκBα to reduce NF-κB and p65 phosphorylation	[43,95]
NSP9	Reduces IFN response via suppression of signal recognition particle (SRP) of the 7SL RNA scaffold Interacts with nucleoporin 62 (a component of nuclear pore complexes) to impede translocation of p65 after TNF-α stimulation	[33,103]
NSP10	Evades IFN-mediated antiviral response by 5′ cap methylation activity via interaction with NSP14 and NSP16 Recognized as a PAMP and stimulates downstream activation of pro-inflammatory cytokines	[120,125]
NSP12	Attenuates type I IFN production by inhibiting IRF3 nuclear translocation Promotes activation of RIPK1 to stimulate ACE2 and EFGR receptors	[43,137,138,139,141]
NSP13	Evades recognition by RLRs Inhibits phosphorylation of TBK1, IRF3, NF-κB Inhibits activation of JAK/STAT signaling	[151,153,154]
NSP14	Works in tandem with NSP16 to evade recognition by MDA5 and RIG-I receptors via mRNA capping Inhibits IFNAR1 expression by targeting the receptor for lysosomal degradation, thus impairing JAK/STAT activation Inhibits host mRNA translational activity to block IFN-I−dependent ISG induction and production of antiviral proteins	[158,161,162]
NSP15	Evades MDA5 immune sensor recognition by cleaving the RNA structure at unpaired uridylate bases in 3′ untranslated regions Attenuates type I and III IFN production by preventing IRF3 nuclear localization Prevents activation of antiviral ISGs	[131,139,178,179,180]
NSP16	Evades recognition by MDA5 and RIG-1 receptors via mRNA capping Inhibits mRNA splicing via interaction with spliceosome components, thus abrogating immune cell development	[33,192]

**Table 3:** Summary of potential inhibitors that target NSP1 to NSP16 of SARS-CoV-2 for antiviral activities.

NSP	Drug Compound	Function	Ref.
NSP1	Montelukast sodium hydrate	Leukotriene receptor antagonist for asthma Binds to C-terminus of NSP1 Reduces viral spike protein, copy number and virus titer	[44]
	Mitoxantrone dihydrochloride	Anticancer drug that can bind to C-terminus of NSP1 Interacts with cell surface heparan sulfate to block viral entry	[45,46]
NSP3	GRL0617	Non-covalent inhibitor against SARS-CoV and SARS-CoV-2 PLpro Upregulates phosphorylation and activation of IFN and NF-κB pathway Inhibits removal of ISG modifications Decreases viral replication and release of viral particles	[57]
	Sitagliptin	Anti-diabetic medication Inhibits PLpro activity Decreases SARS-CoV-2 replication by binding to the catalytic site	[60]
	Daclastavir	Inhibitor of NS5A protein of hepatitis C virus (HCV) Inhibits PLpro by targeting the allosteric site Decreases SARS-CoV-2 replication	[60]
NSP5	Vinyl sulfone (2CN115)	Small molecule NSP5 inhibitor Restores RIG-I expression and induction of ISGs and IFN by binding to the catalytic residues of cysteine protease Reduces NSP5-mediated RIG-I cleavage and production of infectious virions	[68]
	Ivermectin	Antiparasitic drug Interacts with the catalytic dyad of NSP5 to hinder its activity	[74]
NSP6	1α,25-dihydroxyvitamin D3	Active form of vitamin D3 Attenuates NSP6-induced autophagic flux impairment, inflammasome activation, pyroptosis, and ROS production	[82]
	Metformin	Anti-diabetic drug Attenuates NSP6-induced autophagic flux impairment, inflammasome activation, pyroptosis, and ROS production	[82]
	Polydatin	Phytochemical agent Attenuates NSP6-induced autophagic flux impairment, inflammasome activation, pyroptosis, and ROS production	[82]
	Dextromethorphan	Sigma receptor agonist Leads to NSP6 destabilization and an increase in conformational dynamics	[75]
	Haloperidol	Anti-psychotic drug Confers stronger binding affinity and favorable molecular interactions with NSP6	[75]
NSP7–12, NSP8–12 complex	Cepharanthine	Anti-cancer, anti-inflammatory, anti-parasitic and antioxidant alkaloid tetrandrine Targets both the NSP7–NSP12 and NSP8–NSP12 interfaces	[87,88]
	Lonafarnib	Non-peptidomimetic inhibitor of farnesyltransferase intended for progeria Interacts with the NSP7–NSP12 interface	[87,90]
	Nilotinib	Small-molecular tyrosine kinase inhibitor Interacts with the NSP7–NSP12 interface	[87,89]
	Filibuvir	Oral non-nucleoside HCV NS5b RNA-dependent RNA polymerase (RdRp) inhibitor Blocks the NSP8–NSP12 interface	[87,91]
	Olysio	HCV NS3/4a protease inhibitor Blocks the NSP8–NSP12 interface	[87,92]
	Saquinavir	HIV protease inhibitor Binds to the NSP7–NSP12 interface of SARS-CoV	[87,94]
	Tipranavir	Non-peptide protease inhibitor Binds to the NSP7–NSP12 interface of SARS-CoV	[87,93]
NSP9	Fluspirilene	Anti-psychotic drug from diphenylbutylpiperidine family to treat schizophrenia	[110,196]
	Troglitazone	Anti-diabetic and anti-inflammatory drug Inhibits acyl-CoA synthetase 4 (ACSL4)	[110,197]
NSP10–16 complex	Tegobuvir	Novel inhibitor of HCV RNA replication	[198]
	Siramesine	Sigma-2 receptor agonist Disrupts lysosomal pH gradient Impacts cellular trafficking through endosome/lysosome endocytic pathways Promotes accumulation of autophagosomes	[199]
	Bemcentinib	Highly selective oral AXL tyrosine kinase inhibitor Possesses anti-inflammatory and anti-fibrotic roles in non-cancer diseases	[200,201]
	Sonidegib	Selective smoothened (SMO) inhibitor Regulates Hedgehog signaling pathway involved in tissue processes and immunity	[202]
	Itacitinib	JAK1-selective inhibitor of the JAK/STAT pathway Modulates distinct cytokine pathways	[203]
NSP13	Cepharathine	Anti-inflammatory drug Targets NSP13 ATP-binding site Prevents production of 5′-ppN end for transfer of guanidine monophosphate by RNA GTase which is critical for formation of primary mRNA capping structure	[150,156]
NSP14	Ritonavir	Inhibits formation of the complex of NSP14 with RNA Prevents excision of nucleotides from the 3′ end of the growing RNA strand	[163]
	SAM analogs (DS0464, SS148)	Competitive inhibitors against S-adenosylmethionine Prevents methyl donation to inhibit NSP14 activity	[165,166]
NSP15	Epigallocatechin gallate, glycyrrhizic acid	Binds to active site of NSP15 Prevents cleavage of RNA nucleotide at the 3′ end of uridylates Dampens viral replication	[181,185]
NSP16	Sinefungin	Pan-methyltransferase inhibitor Binds to the SAM binding pocket of the NSP10-NSP16 complex Prevents mRNA capping that contributes to immune evasion	[118,204]
	Maraviroc, raltegravir	Approved HIV drugs Considerable in silico binding activity to SARS-CoV-2 NSP16 binding pocket Prevents mRNA capping	[194,195]

## 3. Conclusions

The rapid emergence and spread of COVID-19 have focused efforts on discovering therapeutic strategies with useful antiviral potential. SARS-CoV-2 NSPs that contribute to host immune escape could hold crucial keys to the success of such endeavors. Notably, NSP1 to NSP16 play significant roles in establishing viral infection in COVID-19. The major NSP-associated immune escape mechanisms are attributed to the capability of these viral proteins to inhibit critical interactions underpinning PAMP recognition by infected host cells—thereby masking the presence of PAMPs. For example, NSPs 3, 8, 13, 15, and 16 evade or dampen MDA5 immune sensors which would otherwise flag non-self or foreign antigens and molecules that concomitantly upregulate type I and III IFNs which in turn induce a cascade of antiviral gene responses. To date, it is fortunate that several drugs (such as remdesivir and paxlovid) have been approved by FDA and other regulatory authorities for clinical management. Such drugs are being deployed as interim SARS-CoV-2 NSP inhibitors to fulfill the pressing need to confront this COVID-19 pandemic. Going forward, more detailed investigations are being carefully considered to elucidate the potential of existing and novel NSP inhibitors against host immune evasion conferred by SARS-CoV-2 NSPs.

## Figures and Tables

**Table 1:** Summary of the structural proteins of SARS-CoV-2 and their functions.

Structural Protein	Functions	Reference
S	Binds to host angiotensin-converting enzyme-2 (ACE2) receptor for fusion with the host cell membrane via endocytosis.	[11]
M	Promotes viral assembly by stabilizing the N protein-RNA complex inside the virion.	[12]
E	Forms the “viroporin” ion channel. Interacts with M protein to form viral particles. Acts on virion release and pathogenesis.	[13]
N	Packages viral genomic RNA into a helical ribonucleoprotein form.	[14]

## Data Availability

Not applicable.
